# Systematic Analysis of the Transcriptome Profiles and Co-Expression Networks of Tumour Endothelial Cells Identifies Several Tumour-Associated Modules and Potential Therapeutic Targets in Hepatocellular Carcinoma

**DOI:** 10.3390/cancers13081768

**Published:** 2021-04-07

**Authors:** Thomas Mohr, Sonja Katz, Verena Paulitschke, Nadim Aizarani, Alexander Tolios

**Affiliations:** 1 ScienceConsult—DI Thomas Mohr KG, Enzianweg 10a, A-2353 Guntramsdorf, Austria or thomas.mohr@meduniwien.ac.at (T.M.); sonja.katz@hotmail.com (S.K.); 2 Institute of Cancer Research, Department of Medicine I, Medical University of Vienna and Comprehensive Cancer Center, A-1090 Vienna, Austria; 3 Department of Dermatology, Medical University of Vienna, A-1090 Vienna, Austria; verena.paulitschke@meduniwien.ac.at; 4 Max-Planck-Institute of Immunobiology and Epigenetics, D-79108 Freiburg, Germany; aizarani@ie-freiburg.mpg.de; 5 Department of Blood Group Serology and Transfusion Medicine, Medical University of Vienna, A-1090 Vienna, Austria; 6 Center of Physiology and Pharmacology, Institute of Vascular Biology and Thrombosis Research, Medical University of Vienna, A-1090 Vienna, Austria; 7 Section of Artificial Intelligence and Decision Support, Center for Medical Statistics, Informatics and Intelligent Systems, Medical University of Vienna, A-1090 Vienna, Austria

**Keywords:** hepatocellular carcinoma, network analysis, tumour associated endothelial cells, liver endothelial cells

## Abstract

**Simple Summary:**

Endothelial cells, the innermost layer of blood vessels, play an essential role in the progression of cancer, particularly liver cancer. To develop cancer therapies targeting those cells, the investigation of gene co-expression networks is of great importance. In this study, we investigated the gene expression profile of tumour endothelial cells. We compared it to endothelial cells from non-tumour liver-tissue. Using gene-network based methods, we could identify genes that may play a unique role in liver cancer progression or be a target for cancer therapy. Additionally, we provided a framework for similar analyses in other cancers.

**Abstract:**

Hepatocellular carcinoma (HCC) is the sixth most common cancer and the third most common cause of cancer-related death, with tumour associated liver endothelial cells being thought to be major drivers in HCC progression. This study aims to compare the gene expression profiles of tumour endothelial cells from the liver with endothelial cells from non-tumour liver tissue, to identify perturbed biologic functions, co-expression modules, and potentially drugable hub genes that could give rise to novel therapeutic targets and strategies. Gene Set Variation Analysis (GSVA) showed that cell growth-related pathways were upregulated, whereas apoptosis induction, immune and inflammatory-related pathways were downregulated in tumour endothelial cells. Weighted Gene Co-expression Network Analysis (WGCNA) identified several modules strongly associated to tumour endothelial cells or angiogenic activated endothelial cells with high endoglin (*ENG*) expression. In tumour cells, upregulated modules were associated with cell growth, cell proliferation, and DNA-replication, whereas downregulated modules were involved in immune functions, particularly complement activation. In *ENG*^+^ cells, upregulated modules were associated with cell adhesion and endothelial functions. One downregulated module was associated with immune system-related functions. Querying the *STRING* database revealed known functional-interaction networks underlying the modules. Several possible hub genes were identified, of which some (for example *FEN1*, *BIRC5*, *NEK2*, *CDKN3*, and *TTK*) are potentially druggable as determined by querying the *Drug Gene Interaction database*. In summary, our study provides a detailed picture of the transcriptomic differences between tumour and non-tumour endothelium in the liver on a co-expression network level, indicates several potential therapeutic targets and presents an analysis workflow that can be easily adapted to other projects.

## 1. Introduction

Hepatocellular carcinoma (HCC) is the sixth most common cancer and the third most common cause of cancer-related death, affecting roughly 500,000 people worldwide each year [1]. It arises in a multistep process from preexisting cellular lesions [2], often aggravated due to malnutrition, increased alcohol consumption and obesity [3]. Chemotherapeutic options are often limited, and prognosis after primarily curative surgery is usually poor [4]. Between 50 and 60% of patients who undergo primary resection with curative intent are experiencing a recurrence of the tumour within five years [5].

Endothelial cells represent roughly 20% of the liver’s overall number of cells and approximately 3% of the liver volume [6,7], and they contribute actively to various processes such as metabolite transport, hemostasis in case of vascular damage, maintenance of vascular tone, inflammation, and angiogenesis [8]. Liver endothelial cells consist of two major subgroups, macrovascular endothelial cells and liver sinusoidal endothelial cells (LSEC), with LSEC presenting the majority of endothelial cells present in the liver. LSEC display fenestrated morphology with minimal basement membrane [9]. A subset of them express high levels of *CD36*, which is generally not detectable on other types of endothelial cells [10], and endoglin (*ENG*), a marker for angiogenic activation [11]. *PECAM1*, *CD34* and classic adhesion molecules like *ICAMs* are weakly expressed [11]. Non-canonical adhesion molecules (*VAP1*, *DESIGN*, *LYVE1*, and *MADCAM*) are present, underlining the role of LSEC in the immune cell recruitment [11]. In unstimulated LSEC, chemokines and cytokines are only comparatively weakly expressed [11].

When tumours coopt endothelial cells during induction of angiogenesis, a switch in endothelial phenotype occurs that is characterised by loss of differentiation, development of drug resistance [12], a higher angiogenic potential [13], and a distinct gene expression pattern [14]. In HCC, this is evidenced by a loss of fenestration, abrogated expression of markers such as *LYVE1* [15], profound angiogenesis, a hyper-vascularised microenvironment [15], and the expression of a cocktail of growth factors and cytokines [11,16]. Thus, tumour endothelial cells play a significant role in at least four of the ten *hallmarks of cancer* [17], namely “Inducing angiogenesis” [16], “Tumor promoting inflammation” [11], “Activating invasion and metastasis” [18,19], and possibly in “Resisting cell death” [15,20], making them promising targets in for HCC therapy. A combination of bevacizumab and atezolizumab is the first-line treatment in advanced HCC [21], highlighting the role of angiogenesis as target for a successful treatment and the clinical relevance to analyze specific features of TECs for potential new therapeutic targets.

However, discovering potential new therapeutic approaches aimed at endothelial cells requires in-depth knowledge about endothelial cells’ contribution to cancer progression, especially concerning underlying molecular networks. Analysis of transcriptomics datasets derived from normal and tumour endothelium using state-of-the-art co-expression network analysis methods are uniquely suitable for this task. Similar approaches have been applied to tumour cells of various cancers, including HCC [22,23,24,25], but to the best of our knowledge never used to compare tumour versus non-tumour endothelium. Network-based methods identify groups of genes - so-called modules - that act similarly across groups of samples as defined, e.g., by clinical traits, cell types, or other parameters [26]. Modules of interest can be identified by measuring the association of the so-called eigengene (a representation of the gene expression pattern of the module) with the trait of interest, in our case cell location (tumour versus non-tumour) and angiogenic activation (using endoglin as an activation marker, therefore comparing *ENG*^+^ versus *ENG*${}^{-}$), respectively. The biological context of these modules can then be investigated by term enrichment analysis, which identifies biological processes of potential relevance for the underlying pathology. Using network topology based measures, it is possible to detect key drivers and potential therapeutic targets in various pathologic processes [26,27,28].

Our study re-analyses a published dataset (E-GEOD-51401/GSE51401) derived from endothelial cells from hepatocellular carcinoma and adjacent non-tumour tissue. First, we assessed the data’s feasibility by analysing endothelial markers and compared them with RNASeq data. Then, we analysed differences in the gene expression profile and the biological context using Gene Set Variation Analysis. Third, we employed co-expression network techniques to detect gene modules associated with cell origin (tumour versus non-tumour tissue) and angiogenic activation (using endoglin as an activation marker, therefore comparing *ENG*^+^ versus *ENG*${}^{-}$ cells) and investigate the biological context of these modules. Forth, we used network-based parameters to determine hub genes and potential key drivers in the detected modules. Finally, we identified potential therapeutic targets by querying the *Drug–Gene Interaction database* with the hub genes as input. This study presents a detailed characterisation of HCC-associated tumour endothelial cells on the transcriptome level and an analysis workflow easily adaptable to other projects.

## 2. Materials and Methods

### 2.1. Data Generation and Access

The wet-lab experiments for GSE51401 were performed by Sun H and Wang X (Zhongshan hospital, affiliated to Fudan University, Shanghai, China) [20]. For a detailed description of the protocol and the experimental design, see the parallel study by Xiong et al. [20]. Briefly, the specimen of tumours (tumour endothelial cells, TEC) or surrounding non-tumour tissue (non-tumour endothelial cells, NEC) were collected after removal from patients with HCC as determined using routine clinical, histopathological analysis as well as positive alpha-fetoprotein (AFP). After washing and homogenisation, *PECAM1*^+^/*ENG*${}^{-}$, as well as *PECAM1*^+^/*ENG*^+^ cells, were isolated using magnetic beads conjugated with antibodies against *PECAM1* and *ENG*. The latter plays a crucial role in angiogenesis via regulation of cell proliferation and migration [29,30] and serves as a marker of angiogenic activation [31]. Thus, the dataset represents quiescent and angiogenic-activated endothelial cells from tumour and non-tumour liver tissue, without differentiation between endothelial subtypes. Gene expression profiling was performed by the ShanghaiBio Corporation (Shanghai, China) using the Human Genome U133 Plus 2.0 Arrays (Affymetrix, Santa Clara, CA, USA) according to manufacturer’s instruction. The authors made the data publicly available via *ArrayExpress*/*Gene Expression Omnibus* as GSE51401 [20].

### 2.2. Data Preprocessing and QC

CEL files were downloaded and read into “GNU R” [32] using the package “affy” [33] and preprocessed using the R-package “arrayQualityMetrics” [34]. Arrays were marked as outliers and excluded from further analysis if they exceeded the outlier thresholds defined by Kauffmann et al. [34] in any of the quality control parameters: the distance between arrays in the principal component analysis, relative log expression, normalised unscaled standard error, joint distribution of M and A, and spatial distribution. Subsequently, arrays were normalised by robust multiarray average (RMA) normalisation using the R-package “affy” [33]. Probes matching to multiple genes, antisense RNAs, pseudogenes, or uncharacterised loci were removed before further analysis, and genes with several probes present on the array were summarised to the probe with the maximal interquartile range using the “genefilter” package [35]. The resulting expression sets were subjected to a second QC analysis and removal of outliers as described above.

### 2.3. Single-Cell RNASeq

Single-cell RNA sequencing (scRNASeq) data were obtained from liver tissue originating from individuals with (3) and without (9) HCC by mCEL-Seq2 as described by Aizarani et al. [36]. After data preprocessing, the resulting expression matrices were clustered and assigned to cell types using the RaceID3 algorithm [37]. Clusters associated with liver sinusoidal endothelial cells, macrovascular endothelial cells and other endothelial cells were used to generate aggregated data for pseudo-bulk RNASeq analysis. Count matrices were read into R and aggregated by gene and patient via summing up counts. Samples were annotated with cell origin (i.e., HCC or non-diseased tissue) and analysed for differentially expressed genes using the R-package “DESeq2” and an expression∼origin design.

### 2.4. Differential Gene and Pathway Expression Analysis

Differential gene expression and pathway enrichment were determined using the R-package “LIMMA” [38]. Cell origin and activation were concatenated to a “group” factor and entered into the model expression∼0+group. The consensus correlation to account for pairing was calculated using the “duplicateCorrelation” function with the patient ID as a blocking variable. The model was fit using the “lmFit” function with the patient ID as a blocking variable and the consensus correlation as inter-duplicate correlation. Finally, moderated t-statistics, F-statistics and log-odds ratio were calculated using the “eBayes” function. *p*-values were adjusted to multiple testing according to Benjamani–Hochberg [39]. The thresholds for differentially expressed genes were set to $|log_{2}\left(FC\right)|>2$ and $p_{adjusted}<0.05$.

### 2.5. Gene Set Variation Analysis

Gene Set Variation Analysis (GSVA) is an extension of the Gene Set Enrichment Analysis for complex experimental designs. Briefly, gene expressions are normalised across samples. Samples are ranked according to the normalised gene expression, and a Kolmogoroff-Smirnov-like rank statistics is calculated for each gene set. This statistics is then translated into a GSVA score, indicating enrichment of genes at the top or bottom of the sample’s ranked gene list. The result is a matrix of GSVA scores, one per gene set and sample, with a positive value indicating overexpression and a negative value indicating underexpression of the geneset’s genes. This approach’s strength lies in the fact that it translates a gene expression matrix to a term expression matrix, which can be analysed using linear models [40]. GSVA was performed using the “gsva” R-package [40]. As input, WikiPathways provided by the CPTAC (Clinical Proteomic Tumor Analysis Consortium, National Institutes of Health, Maryland, Bethesda) portal were used [41]. This group maintains a collection of cancer-related pathways based on cancer hallmarks proposed by Weinberg et al. [17] and encompasses following pathway groups: *Activating invasion and metastasis*, *Avoiding immune destruction*, *Deregulating cellular energetics*, *Enabling replicative immortality*, *Evading growth suppressors*, *Genome instability and mutation*, *Inducing angiogenesis*, *Resisting cell death*, *Sustaining proliferative signalling*, and *Tumor promoting inflammation*. Differentially enriched pathways were determined using the R-package “LIMMA” as described above.

### 2.6. Weighted Gene Co-Expression Network Analysis (WGCNA)

WGCNA was performed according to Langfelder et al. [42], modified by an iterative network-stabilising algorithm as described by Greenfest-Allen et al. [43]. This approach is characterised by pruning genes with a low module membership (calculated distance < 0.8) and repetition of module detection until the network stabilises. A two-Gaussian filter approach was used to remove noise due to lowly expressed genes. This approach assumes that the distribution of gene-expression follows two Gaussians, one at the lower end of the expression range representing probes with low expression values (and presumable background expression) and the other representing probes with high expression values. A threshold can be chosen so that genes with an expression value above this threshold have a higher probability of belonging to the Gaussian representing expressed genes. The threshold was set using the R-package “mixtools” [44]. Genes with a log2 expression values below the set threshold of 5.33 in all samples were excluded from further analysis. Potential outliers were removed by calculating the z-normalized inter-sample connectivity (zK) according to Oldham et al. [45]. Samples showing an ∣zK∣>1.96 were removed prior to further analysis.

WGCNA was run using the parameters as suggested by Greenfest-Allen et al. [43] for an iterative WGCNA approach except for the following: network type: “signed”, β: 12, deepsplit: 2, correlation: “bicor”, and pamStage: TRUE. A signed network approach was used to focus on the positive correlation between the genes. The goodness-of-fit of the modules was estimated by determining the median module membership of the genes, defined as the absolute correlation between the gene and the module eigengene. It can be used to assess the goodness-of-fit of the modules to the eigengenes. A module membership of 1 denotes a perfect fit, whereas a value of 0 shows no fit. Only modules with a median module membership of greater than 0.8 were considered for further analysis. The association of modules with cell origin or angiogenic activation was determined by fitting a mixed-effects model (using the R-package “lme4” [46]) and calculating the t-statistics as described by Li et al. [47]. The t-statistics can be used to assess the strength of the association between a module and a sample trait [47]. Following model was used to assess module association with cell origin or angiogenic activation:

$$y_{ijk}=\beta_{0i}+\beta_{1}\cdot origin_{j}+\beta_{2}\cdot activation_{k}+\epsilon_{ijk}\;i=1,\ldots ,n,\;j=1,2,\;\mathrm{and}\;k=1,2$$

where $y_{ijk}$ is the expression level of the eigengene of the *i*-th subject and the *jk*-th sample, $origin_{j}$ is the cell origin (with *j* = 1 denoting non-tumour and *j* = 2 denoting tumour tissue), $activation_{k}$ is the activation status (*k* = 1 denoting *ENG*${}^{-}$ and *k* = 2 denoting *ENG*^+^ cells), $\beta_{0i}$ is the subject specific random intercept to account for pairing, and $\epsilon_{ijk}$ the random error term [47]. The t-statistics and the associated *p*-value was calculated using the R-package “lmerTest” [48]. *p*-value correction was done according to Benjamani–Hochberg using the “p.adjust” function in the R-package “stats” (method = “hochberg”).

Hub genes were determined by calculating the intramodular connectivity (*kWithin*) [26], normalized to the maximum connectivity within the module for each gene (*kWithin.norm*) according to the following model:

$$kWithin.norm_{i}=\frac{\sum_{i\in module}a_{i}}{max(\sum_{i\in module}a_{i})}$$

where *a* is the adjacency measure used to calculate the co-expression network. Genes strongly correlating with many other genes within the module will get high intramodular connectivity, whereas genes with weak correlations will receive low intramodular connectivity. The top ten percent of genes in terms of intramodular connectivity were considered as potential hub genes. To map strong correlations between genes with known functional interactions, the *STRING database* was queried using the “STRINGdb” R-package [49].

To determine potential druggability of identified hub genes, we performed a query on the *Drug–Gene Interaction database* (DGIdb) using an in-house developed API querying function [50,51].

### 2.7. Term Enrichment Analysis for Detected Modules

To determine the biological context of the modules, GO term Enrichment Analysis was done using the R-package “clusterProfiler” [52] querying the *Gene Ontology—Biological Process* database [53,54]. According to the concept that the background of a term enrichment analysis should encompass all genes interrogated, all genes present in the final expression set were defined as background. Only terms containing at least 15 genes and at most 500 genes were considered. Terms with an adjusted *p*-value (Benjamani–Hochberg) of less than 0.05 were considered to be significantly enriched. Terms smaller than fifteen or larger than 500 genes were excluded from the analysis. GO term reduction was done using a GOTSSA (GO term Semantic Similarity Analysis) approach. Term lists were loaded into R using the “GOSemSim” package to calculate semantic similarity based on the method described by Wang et al. [55,56]. The dimensionality of the resulting similarity data was reduced by Uniform Manifold Approximation and Projection (UMAP) [57] using the “umap” function of the “M3C” package and segmented into clusters by density-based clustering using the “fpc” R-package with an ϵ of 0.6 and 5 as a minimum number of neighbours [58]. The GO term with the highest $-log10(p_{adusted})$ was chosen as a representative for the respective cluster [59].

### 2.8. Statistics

If not stated otherwise, comparisons between groups were performed using Student’s *t*-test. If necessary, *p*-values were adjusted for multiple testing using the Benjamani–Hochberg procedure [39]. An adjusted *p*-value below 0.05 was considered to be statistically significant.

## 3. Results

**Figure 1:**
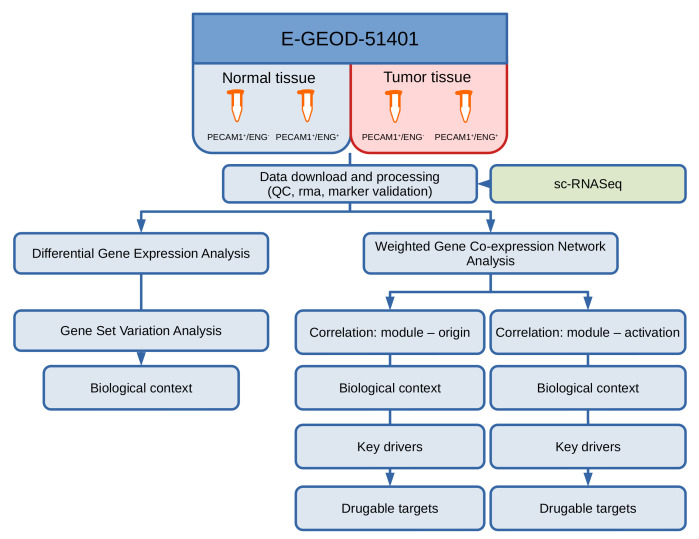
Workflow of the overall analysis.

After downloading the raw data, preprocessing and performing quality control, differences in the gene expression profiles were determined using the R-package “LIMMA” and put into biological context by performing Gene Set Variation Analysis. A co-expression network was constructed using WGCNA and segmented into groups of genes (so-called modules) with similar co-expression characteristics. The top two modules’ biological context with a high positive and the top module with a strong negative association to cell origin or angiogenic activation was investigated by term enrichment analysis. Finally, the *Drug–Gene Interaction database* (*DGIdb*) was queried using hub genes (as determined by their connectivity within the module) to determine potential drug targets (Figure 1).

### 3.1. Cohort Characteristics

Data preprocessing and quality control resulted in a dataset with 43 samples, drawn from 16 subjects (3 females, median age 64; 13 males, median age 52). The population characteristics, original samples and excluded samples are shown in Table 1. The discrepancy between the number of *ENG*${}^{-}$ and *ENG*^+^ samples is due to repeated sampling of the *ENG*${}^{-}$ fraction.

### 3.2. The Expression Pattern of Endothelial Markers Is in Accordance with That of Liver Endothelial Cells

**Figure 2:**
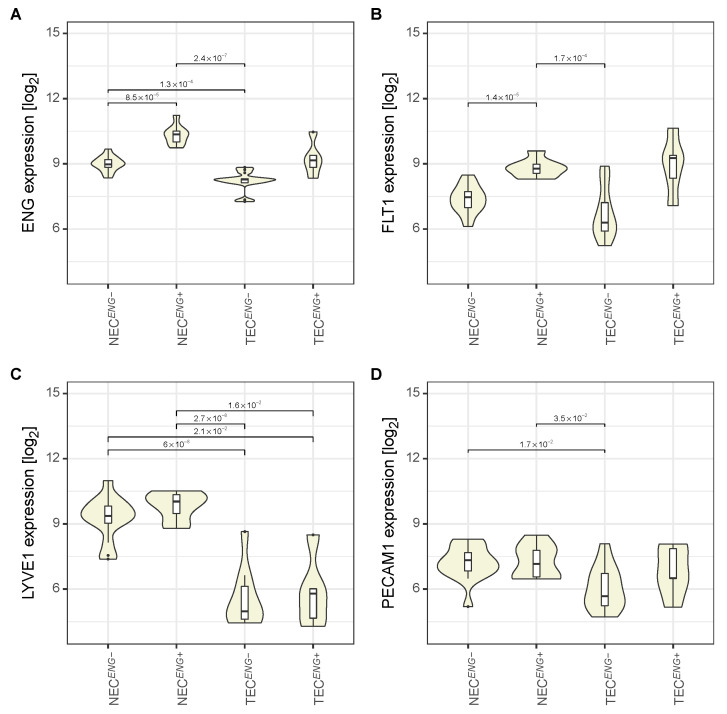
Expression of *ENG*, *FLT1*, *LYVE1*, and *PECAM1* in TEC and NEC (*ENG*${}^{-}$ and *ENG*^+^). Violin plots of the expression distribution of (**A**) *ENG*, (**B**) *FLT1*, (**C**) *LYVE1*, and (**D**) *PECAM1* are shown. Numbers above the bars represent adjusted *p*-values of the comparisons indicated by the bar.

To test the data quality, the expression of LEC markers *PECAM1*, *ENG*, *LYVE1*, and *FLT1* was investigated across all groups. All genes displayed an expression pattern as described previously [7]: expression of *ENG*, *PECAM1*, *LYVE1* and *FLT1*, and upregulation of *ENG* in *ENG*^+^ NEC (Figure 2A–D). TEC displayed a trend (non-significant, adjusted *p*-value = 0.17) to *ENG* upregulation in *ENG*^+^ cells (Supplementary Table S1). The lower expression of all markers in TEC compared to NEC is in line with the reported de-differentiation in these cells [15].

**Figure 3:**
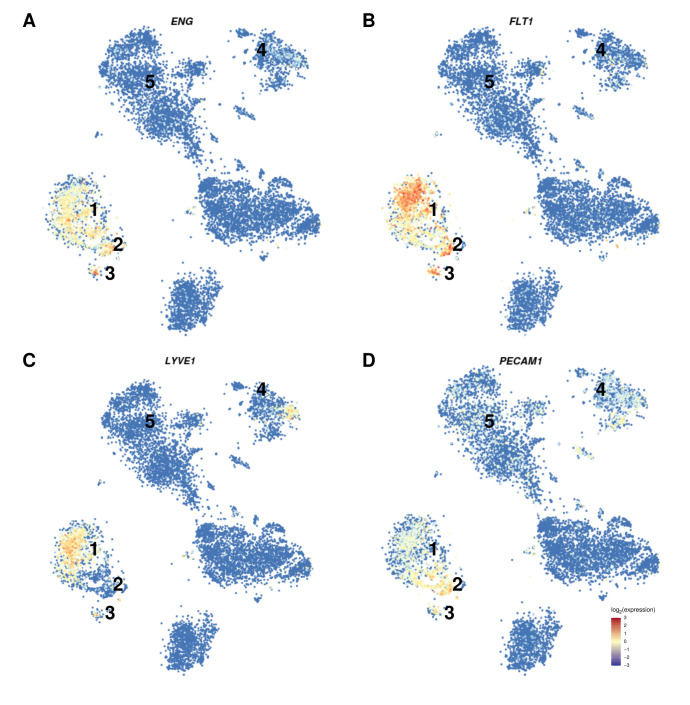
Expression of *ENG*, *FLT1*, *LYVE1*, and *PECAM1* in liver cells. The distribution of the expression of (**A**) *ENG*, (**B**) *FLT1*, (**C**) *LYVE1*, and (**D**) *PECAM1* in sc-RNASeq cluster of liver cells is shown. The cluster association is as follows: (1) Liver sinusoidal endothelial cells, (2) macrovascular liver endothelial cells, (3) other liver endothelial cells, (4) Kupffer cells, (5) NK, NKT, and T-cells. Expression levels are color-coded from blue to red as indicated at the lower right corner.

It has been reported that *PECAM1* is also expressed in Kupffer and hematopoietic cells, thus leading to potential contamination of the isolate [60]. Therefore, we investigated liver single-cell RNASeq data concerning the expression of *ENG*, *FLT1*, *LYVE1*, and *PECAM1*. In single-cell RNASeq analysis, *ENG* and *FLT1* expression were restricted to LEC clusters, with *ENG* being also lowly expressed in Kupffer cell-associated clusters (Figure 3A,B). *LYVE1* expression was restricted to the cluster associated with LEC, with a small cluster of Kupffer cells being *LYVE1* positive (Figure 3C). *PECAM1* showed a high expression in macrovascular endothelial cell-associated clusters, a lower expression in LEC associated clusters and a low to moderate expression in Kupffer cell, NK, NKT, and T-cell associated clusters (Figure 3D). These findings indicate that the use of *ENG* and *PECAM1* for isolation of LEC might lead to contamination of the isolate with Kupffer, NK, NKT and T-cells (Figure 3A,D, clusters 4 and 5) [60]. To estimate potential contamination with those cell types, the expression of *PTPRC* (*CD45*), a marker strongly expressed in Kupffer cells and leukocytes in general (therefore also known as leukocyte common antigen, *LCA*), but only weakly expressed in a subset of LEC [36] was determined. The mean expression level of *PTPRC* in GSE51401 was 5.6, which is slightly above our cutoff for lowly expressed genes (5.33). Taken together, these results demonstrate that the gene expression pattern of GSE51401 is consistent with liver endothelial cells.

### 3.3. The Gene Expression Profile of Tumour Endothelial Cells Is Characterised by Upregulated Evasion from Growth Suppressors, Downregulated Immune and Inflammation-Related Pathways, and Resistance to Apoptosis

**Figure 4:**
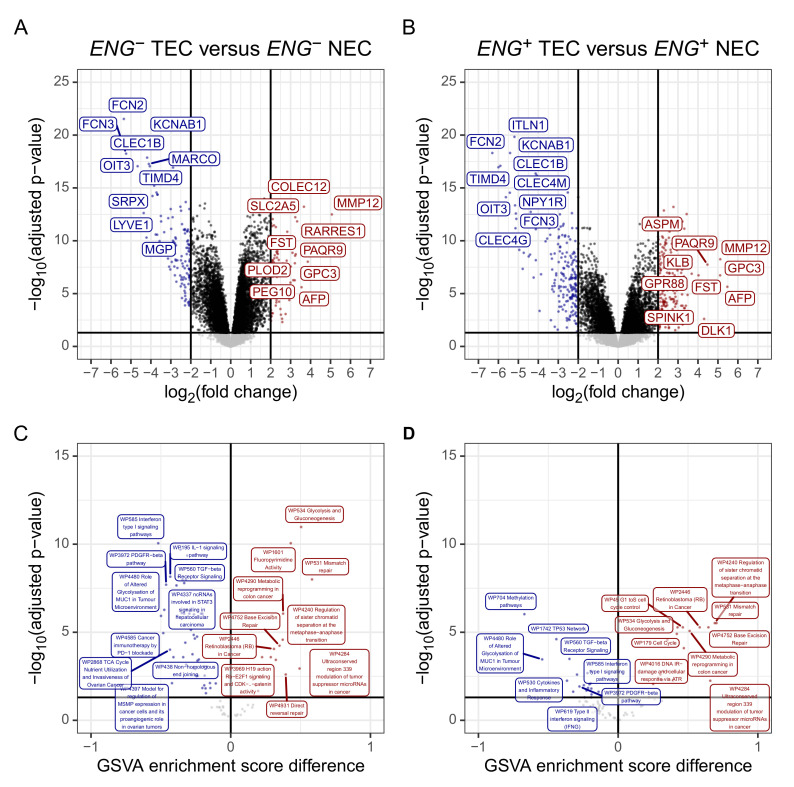
Differential gene expression and pathway perturbation in *ENG*${}^{-}$ and *ENG*^+^ TEC. (**A**) Differentially expressed genes in *ENG*${}^{-}$ TEC and (**B**) in *ENG*^+^ TEC. (**C**) Perturbed Wikipathways in *ENG*${}^{-}$ TEC and (**D**) in *ENG*^+^ TEC. Negative GSVA score difference (blue) indicates down-, positive GSVA score difference (red) indicates upregulation of the respective pathway in TEC. Horizontal lines indicate an FDR of 0.05. The top ten up and down-regulated genes (in term of log2FC) and pathways (in terms of GSVA score difference) are labelled. The controls for *ENG*${}^{-}$ TEC and *ENG*^+^ TEC were *ENG*${}^{-}$ NEC and *ENG*^+^ NEC, respectively.

In *ENG*${}^{-}$ TEC, 223 genes were differentially expressed, with 96 being up- and 127 being downregulated (Figure 4A), 395 were differentially expressed in *ENG*^+^ TEC, with 196 being up- and 199 being downregulated in *ENG*^+^ TEC (Figure 4B). Analysis of pseudo-bulk single-cell RNASeq data confirmed downregulation of. However, only four of the top upregulated genes could be detected in the single-cell RNASeq data. All showed only low expression and could therefore not be used to determine regulation. Single-cell RNASeq, however, has a high gene dropout rate due to technical limitations when performing single-cell experiments, such as RNA degradation during library preparation and limited per-cell sequencing depths [61]. The LIMMA between the *ENG*^+^ and *ENG*${}^{-}$ in TEC and NEC showed 239 differentially expressed genes in NEC (*ENG*^+^ compared to *ENG*${}^{-}$) and 17 differentially expressed genes in TEC (*ENG*^+^ compared to *ENG*${}^{-}$, Supplementary Table S1). Eleven of the 17 differentially expressed genes were differentially expressed in both NEC and TEC. The greater similarity between *ENG*^+^ TEC to *ENG*${}^{-}$ TEC compared to their counterparts from non-tumour tissue may indicate an angiogenic activation of *ENG*${}^{-}$ TEC, which is in line with the literature [13]. The complete results of the LIMMA analysis are available in Supplementary Table S1.

To avoid reliance on arbitrary threshold selection, GSVA, which utilises the entire feature space without prior filtering, was employed in the downstream analysis. Out of 87 pathways, 17 were up and 49 were downregulated in in *ENG*${}^{-}$ TEC (Figure 4C). Upregulated pathways were associated with three *hallmarks of cancer*: “Deregulation of cellular energetics” (for example, *Glycolysis and Gluconeogenesis*), “Evading growth suppressors” (for example, *Cell Cycle* and *Retinoblastoma (RB) in cancer*), and “Genome instability and mutation”. Pathways associated with all other hallmarks of cancer (“Resisting cell death”, “Avoiding immune destruction”, “Sustaining growth signalling”, “Angiogenesis”, and “Tumor promoting inflammation”—*Chemokine signalling pathway*) were mostly consistently downregulated. Interestingly, pathways associated with “Sustaining proliferative signalling” (for instance, *MAPK signalling* and *Ras signalling* were downregulated as well (Supplementary Table S2).

In *ENG*^+^ TEC, out of 87 perturbed pathways, were up and 20 were downregulated (Figure 4D). Again, upregulated pathways were primarily associated with “Deregulation of cellular energetics”, “Evading growth suppressors” (for example *Cell Cycle*), and “Genome instability and mutation”. However, one “Sustaining proliferative signalling”-associated pathway was also upregulated, namely *enhancement of MAP/ERK signalling in diffuse large B-cell lymphoma* (Supplementary Table S2). In *ENG*${}^{-}$ TEC the *Cell cycle* pathway shows several upregulated key genes, the Cyclin group (*CCNB1*, *2*, and *3*) and *CDK1*. Inhibitors regulating DNA damage checkpoints were downregulated (for example, PRKDC). Activators promoting DNA biosynthesis were upregulated (for example, MCMs, Supplementary Figure S1A,B). The log2 fold change ratio of *CCNB1*, *2* and *CDK1*, but not *CCNB3*, was increased even more in *ENG*^+^ TEC. The entry from DNA damage checkpoints (for example, *PRKDC*) was upregulated as well in *ENG*^+^ TEC (Supplementary Figure S1B). *Retinoblastoma gene in cancer*, another pathway associated with “Evasion of growth suppressors”, was also upregulated, with strong overexpression of pathways members leading to G1/S transition (*CCNB1*, *CDK1*) and mitotic spindle association (TTK) (Supplementary Figure S2). Interestingly, *RB1* itself was only weakly overexpressed (Supplementary Figure S2). The *MAPK signalling pathway*, a pathway associated with “Sustaining proliferative signalling”, was found to be downregulated, despite upregulation of several growth factor receptors (for example, *EGFR*, *FGFR3*, and *FGFR4*). The cytokine receptors at the entry of this pathway, however, were found to be downregulated (Supplementary Figure S3) These results indicate that the pro-angiogenic gene expression profile of TEC may depend on the hallmark “Evasion of growth suppressors” rather than sustained “Sustaining proliferative signalling”, or “Angiogenesis”.

In the *Chemokine signalling pathway*, the cytokine-cytokine receptor interaction ligands were mostly upregulated (for example, *CXCL10*, *CXCL9*, and *CXCL5*). *CXCL12*, however, was strongly downregulated. The respective receptors (for instance, *CXCR2*, *CXCR6*, and *XCR1*) were generally downregulated, resulting in a downregulation of the entire pathway (except for *SHC1*, *2*, *3*, and *4*, and *HRAS* and *NRAS* (Supplementary Figure S4A). This regulation pattern was also present in *ENG*^+^ TEC, with a larger log2FC ratio in several ligands (for example, *CXCL13* or *CCL20*) (Supplementary Figure S4B). Taken together, the observed downregulation of the receptors and downstream genes in the *Chemokine signalling pathway* may be a contributing factor to tumour endothelial anergy (insensitivity to pro-inflammatory signals) as described in the literature [62].

In the *Apoptosis* pathway, *BIRC5* and *HELLS* were strongly upregulated in TEC compared to NEC. The logFC ratio of both genes was larger in *ENG*^+^ TEC (Supplementary Figure S5). The complete results of the GSVA are available in Supplementary Table S2.

### 3.4. Weighted Gene Co-Expression Network Analysis Reveals Several Gene Modules Associated Strongly with Cell Origin and Angiogenic Activation

**Figure 5:**
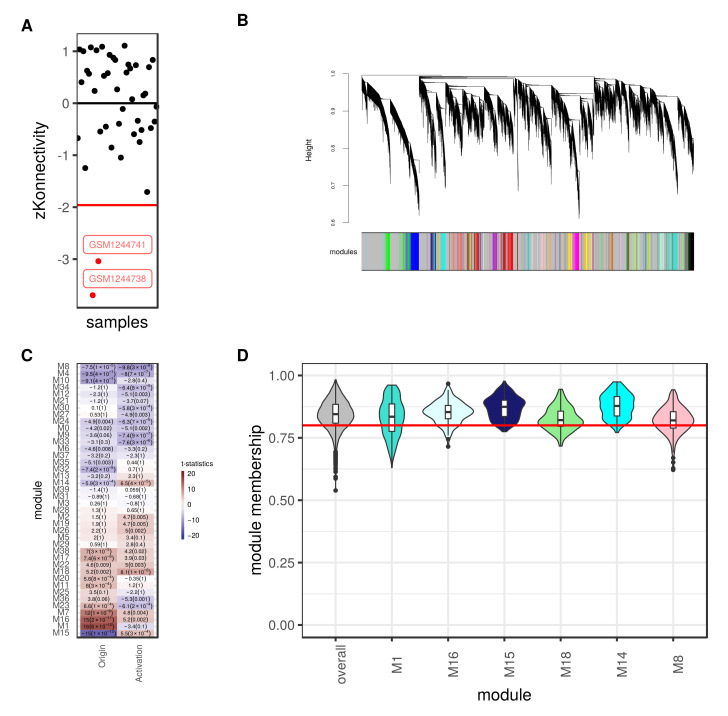
Identification of modules that are associated with either origin (tumour vs. non-tumour endothelial cells) or angiogenic activation. (**A**) Outlier identification based on the z-normalized intersample connectivity (zK). The red line denotes a zK of 1.96. Potential outliers are labelled red. (**B**) Dendrogram showing detected modules. Each branch of the dendrogram represents a gene assigned to one of the modules. Grey areas represent genes not assigned to any module. (**C**) Heatmap showing the association between a module and either cell origin or angiogenic activation. The top number represents the t-statistic, the number in brackets the associated adjusted *p*-value. A positive t-statistics indicates up-, a negative t-statistics represents downregulation in the TEC (indicated by “cell origin”), and *ENG*^+^ cells (indicated by “activation”). (**D**) Module membership for the entire network (overall), for the M1, M16, M15, M18, M14, and M8 module. The red line denotes a membership of 0.8.

Before WGCNA, samples with a zKonnectivity of less than −1.96 were excluded from analysis (Figure 5A). Next, probes representing genes with low expression levels across all samples were excluded from analysis, yielding a co-expression network of 14,690 genes. The iterative approach described by Allen-Greenfest et al. [43] led to the detection of 39 modules with a size between 33 and 433 genes (Figure 5B). The module detection (assigned module and gene connectivity) are provided in Supplementary Table S1 for all genes.

After clustering and module detection (Figure 5B), the association between the cell origin (tumour vs. non-tumour tissue), angiogenic activation (*ENG*^+^ versus *ENG*${}^{-}$ cells), and the module eigengenes was calculated(Figure 5C). Based on module association the M1, M16, M15, M18, M14, and M8 modules were selected for further analysis. All selected modules displayed a median module membership of greater than 0.8 (Figure 5D). This high median module membership is indicative of a stabilised module assignment for the genes of the selected modules.

**Figure 6:**
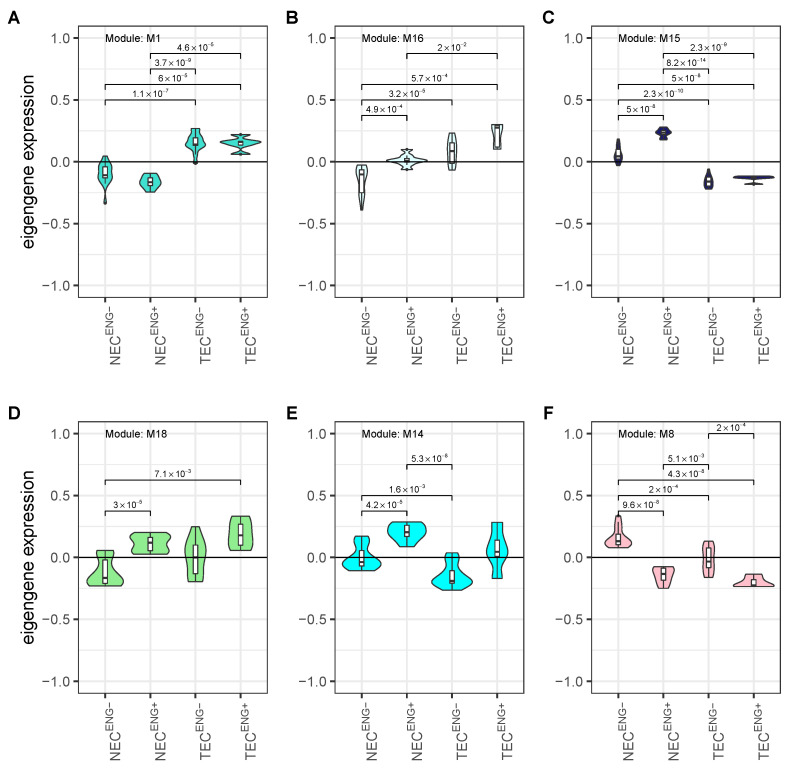
Eigengene plots of modules with strong association to cell origin and angiogenic activation. (**A**–**C**) Eigengene plots for modules with a strong association to cell origin (M1, M16, and M15). (**D**–**F**) Eigengene plots for modules with a strong association to angiogenic activation (M18, M14, and M8. Plots are coloured according to the module colour. Numbers represent adjusted *p*-values for the respective comparisons.

In the M1 module, the eigengenes in *ENG*${}^{-}$ and *ENG*^+^ TEC are upregulated compared to the respective NEC. No difference is observed between *ENG*^+^ and *ENG*${}^{-}$ cells of one origin (Figure 6A). In contrast, the eigengenes in the M16 module show a small but significant upregulation between *ENG*${}^{-}$ and *ENG*^+^ NEC. In *ENG*${}^{-}$ TEC, genes are upregulated compared to *ENG*${}^{-}$ NEC, but not compared to *ENG*^+^ NEC. *ENG*^+^ TEC show a further upregulation of the eigengenes (Figure 6B). This upregulation is not significant compared to *ENG*${}^{-}$ TEC but significant compared to *ENG*^+^ NEC. In the M15 module, *ENG*^+^ NEC show significant upregulation of the eigengenes compared to *ENG*${}^{-}$ NEC. In both *ENG*${}^{-}$ and *ENG*^+^ TEC, the eigengenes are downregulated compared to the respective NEC (Figure 6C). In module M18, eigengene expression is upregulated in *ENG*^+^ TEC and NEC compared to *ENG*${}^{-}$ NEC. both in NEC and TEC. However, eigengene expression in *ENG*${}^{-}$ TEC is slightly but not significantly upregulated in *ENG*${}^{-}$ TEC compared to *ENG*${}^{-}$ NEC. Thus, cell origin’s influence on eigengene expression is small (Figure 6D). In module M14, eigengene expression is upregulated in *ENG*^+^ NEC and downregulated in *ENG*${}^{-}$ TEC compared to *ENG*${}^{-}$ NEC. Interestingly, eigengene upregulation between *ENG*^+^ and *ENG*${}^{-}$ TEC showed only a trend but failed to reach significance (Figure 6E). In module M8, eigengene expression is downregulated in *ENG*^+^ NEC and *ENG*^+^ TEC compared to the respective *ENG*${}^{-}$ cells. In this module, cell origin seems to play a role in eigengene expression since a distinct downregulation can be seen in *ENG*${}^{-}$ TEC compared to the *ENG*${}^{-}$ NEC. Interestingly, *ENG*^+^ TEC showed the same eigengene expression level as *ENG*^+^ NEC. This may reflect phenotypical similarities between TEC and angiogenic activation in NEC as has been reported previously [63] (Figure 6F).

### 3.5. Cell Origin Related Modules Are Positively Associated with Cell Growth and Survival and Negatively Associated with Immune Functions

**Figure 7:**
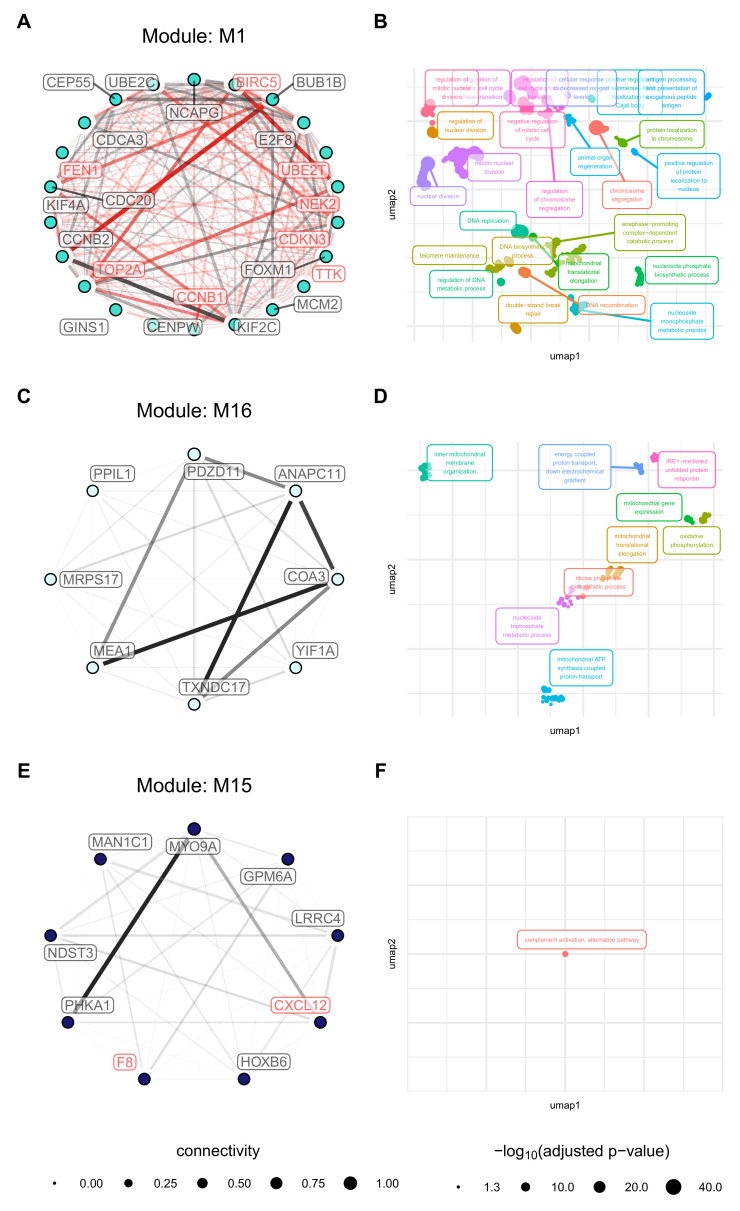
Network and GO Term enrichment plots the hub genes of modules with strong association to cell origin. Network plots (**A**,**C**,**E**) are coloured according to the original module color. Read labels indicate druggability, and edge thickness represents connection strengths (red indicating known FI). GO term Enrichment plots (**B**,**D**,**F**) are coloured according to term clusters and labelled with the most significant term of the respective clusters.

The biological context of the M1 module was defined by cell proliferation-related GO terms (for example, *DNA replication*, *Mitotic nuclear division*, and *Chromosome segregation*). The module contains 29 hub genes, with *BIRC5*, *UBE2T*, *NEK2*, *CDKN3*, *TTK*, *CCNB1*, *TOP2A*, *FEN1*, *AURKA*, *RNASEH2A*, and *POLE2* being druggable (Figure 7A,B). Querying the *STRING database* revealed a dense functional interaction network (FI) between the hub genes, for instance, *BIRC5*, *FEN1*, *NEK2*, *TTK*, *CNB1*, and *CDKN3*, pointing towards an existing FI network underlying this module. It appears that especially the interaction *BIRC5* ⇔ *TOP2A* ⇔ *NEK2* determine module function since the adjacencies between these genes are particularly strong and branch into several other nodes, such as *CDKN3*, *UBE2T*, and *CCNB1*. All of these genes have been proposed as potential drug targets in other cancer entities. The biological context of M16 the module was defined by cell metabolism-related GO terms (*oxidative phosphorylation*) and mitochondria-related GO terms (*mitochondrial translational elongation* and *inner mitochondrial membrane organisation*). Eight hub genes could be detected (*PDZD11*, *ANAPC11*, *COA3*), *YIF1A*, *TXNDC17*, *MEA1*, *MRPS17*, and *PPIL1*. No known functional interactions exist and none of these hub genes was found in the *DGIdb* (Figure 7C,D). In the M15 module, nine hub genes are present (*MYO9A*, *GPM6A*, *LRRC4*, *CXCL12*, *HOXB6*, *F8*, *PHKA1*, *NDST3*, and *MAN1C1*. Of these, *CXCL12* and *F8* are druggable. Querying the *STRING database* showed no known FI between these genes. Biologically the module is characterised by *complement activation, alternative pathway* (Figure 7E,F).

### 3.6. Activation Related Modules Are Associated with Angiogenesis, Cell Adhesion and Immunologic Functions

**Figure 8:**
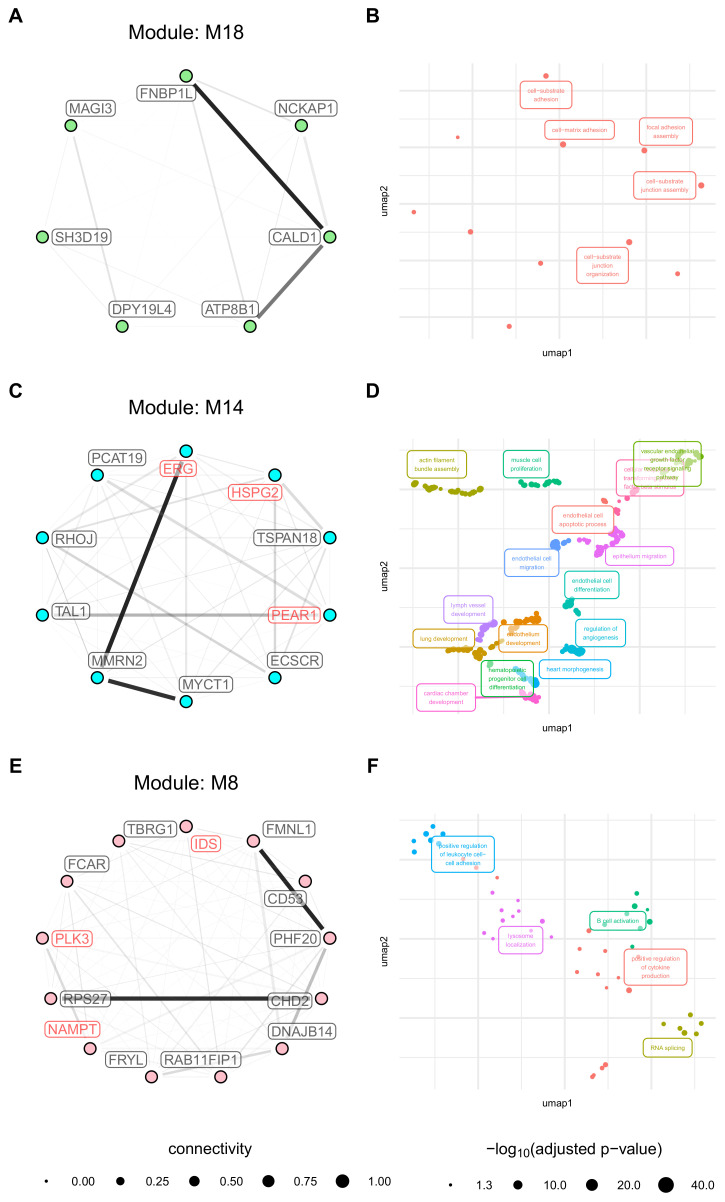
Network and GO Term enrichment plots the hub genes of modules with strong association to cell origin. Network plots (**A**,**C**,**E**) are coloured according to the original module color. Read labels indicate druggability, and edge thickness represents connection strengths (red indicating known FI). GO term Enrichment plots (**B**,**D**,**F**) are coloured according to term clusters and labelled with the most significant term of the respective clusters.

The biological context of the M18 module was defined by *cell-matrix adhesion* and *focal adhesion assembly*. The module contains seven hub genes, none of which are druggable (Figure 8A,B). Several endothelial cell GO term clusters, for example, *vascular endothelial growth factor receptor signalling pathway*, *endothelial cell migration*, and *endothelial cell differentiation*, define the biological context of the M14. It further contains 10 hub genes, none, however, being drugggable (Figure 8C,D). Finally, the GO term clusters detected in the M8 module are associated with cytokine production (*Positive regulation of cytokine production*) and immune functions (*B cell activation*) and cell migration (*positive regulation of leukocyte cell-cell adhesion*). It contains 13 hub genes, with *IDS*, *NAMPT*, and *PLK3* being druggable (Figure 8E,F). Despite a strong adjacency between several modules’ hub genes, particularly the M8 module, no known FI have been found. Co-expression networks, however, indicate not only direct functional interactions but also interactions covering several intermediate proteins. The strong adjacencies might indicate that these genes are part of a cross pathway network. The complete GO term enrichment analysis results for all detected modules are provided in Supplementary Table S3.

### 3.7. Modules with a Strong Positive Association with Cell Origin and Angiogenic Activation Contain Several Potentially Druggable Hub Genes

Several hub genes of modules positively associated with cell origin and angiogenic activation could be identified, namely *BIRC5*, *UBE2T*, *NEK2*, *CDKN3*, *TTK*, *CCNB1*, *TOP2A*, and *FEN1* for the M1 module, and *ERG*, *HSPG2*, *PEAR1*, *TIE1*, *IDS*, *NAMPT*, and *PLK3* for the M18, M14, and M8 modules. A number of drugs targeting these genes are already under investigation, especially in connection with *FEN1*, *BIRC5*, and *NEK2* [64]. The associated drugs and PMIDs are listed in Table 2.

**Figure 9:**
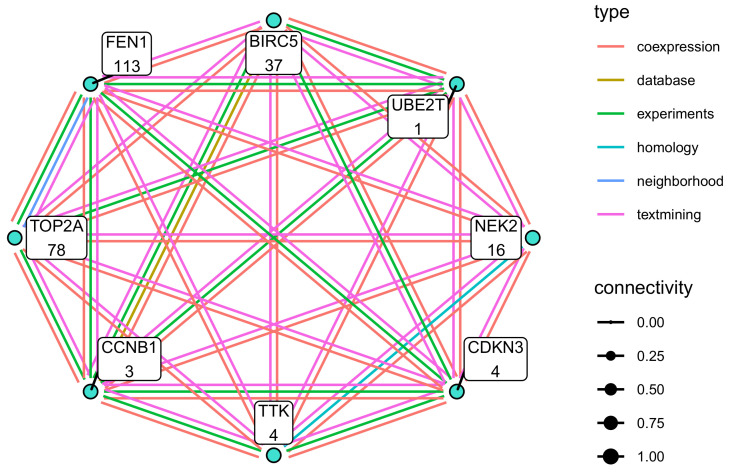
Functional Interaction network underlying the potentially druggable hub genes in module M1. The functional interaction network between potentially druggable hub-genes is shown. Edge color represents interaction type. The number indicates the number of drugs targeting the gene.

The M1 module contained several hub genes interconnected by a known functional interaction network that can be targeted by several drugs (Figure 9). The majority of the functional interactions have been inferred from co-expression, database, and text mining approaches. The interactions *BICR5* ⇔ *UBE2T* ⇔ *FEN1* ⇔ *CCNB1* ⇔ *CDKN3* ⇔ *TTK* was confirmed by experimental evidence either in humans or in homologs from other organisms. These findings suggest that a regulatory network may underly module M1. The complete results of the drug-gene interaction database query are provided in Supplementary Table S4.

## 4. Discussion

Tumour endothelial cells play a decisive role in cancer progression. Numerous studies have been undertaken to investigate the underlying biological processes and identify prognostic markers or therapeutic targets in HCC and other tumour entities [36,65,66,67,68]. However, except for the dataset used in this study (GSE51401), these studies are of too limited size to allow the systematic integration of differential gene expression and pathway perturbation analysis with advanced bioinformatics approaches. Consequently, they focused on differentially expressed genes, resulting in a general picture of perturbed biologic functions. Nowadays, advanced network-based bioinformatics approaches such as Weighted Gene Co-expression Network Analysis (WGCNA) allow gaining a more in-depth insight into the biological context of expression data by identifying gene modules with similar expression patterns across conditions and identifying hitherto unknown gene-gene correlation networks, hub genes, and potential therapeutic targets.

Our study combined differential gene expression analysis, GSVA and WGCNA with querying the *Drug–Gene Interaction database* and *STRING database* to re-analyze GSE51401, a dataset containing TEC and NEC derived transcriptomics data. We identified differentially regulated genes, pathways, gene modules and their biologic functions, hub genes, and potential therapeutic targets.

Our results show that the pro-growth phenotype reported for the TEC for this dataset [20] as well as in other studies [69] may be caused by pathways involved in “Evading growth suppressors” rather than “Sustaining proliferative signalling” or “Inducing angiogenesis” patterns. Somewhat surprisingly, the *Angiogenesis* and *MAPK signalling pathway* pathways were downregulated despite upregulation of the main ligands (*VEGFA* and *PDGFB*) and receptors (*EGFR*, *FGFR3*, and *FGFR4*). The fact that almost all “Sustaining proliferative signalling” and “Inducing angiogenesis” related pathways were consistently downregulated in TEC leads to the conclusion that the sustained cell growth observed in TEC may be driven by signals stemming from other sources. These results raise questions about the origin of these signals and may have implications for the design of anti-angiogenic therapies. Tumour endothelial cells may develop apoptosis resistance [70], as has been demonstrated in the cells of GSE51401 [20]. Our analysis indicates that BIRC5/Survivin and HELLS’s overexpression, two apoptosis inhibitors, may be instrumental in conferring apoptosis resistance in TEC. In the *Chemokine signalling pathway*, a panel of ligands was upregulated, for example, *CXCL5*, *CXCL11*, *CXCL9*, and *CXCL10.* The receptors, for example, *CXCR2*, were generally downregulated in *ENG*${}^{-}$ and *ENG*^+^ TEC. Particularly *CXCR2* is a receptor for angiogenic cytokines [71] and thought to mediate pro-angiogenic signalling [72]. This was again somewhat surprising since upregulation of chemokine receptors has been observed in several other tumour entities and is thought to be supporting TEC [73].

The second result of our study is the identification of modules associated with tumour and angiogenic activated EC. The M1 module was strongly associated with cell cycle-related functions and contained 22 hub genes, for example, *FEN1B*, *BIRC5*, *UBE2T*, *NEK2*, *CDKN3*, *TTK*, *CCNB1*, and *TOP2A*. This is confirmed by an HCC-associated network proposed by Xing et al. [74], which was derived from five studies in HCC. This network contains twelve of our hub-genes and has BIRC5 as the central gene as well. The adjacencies between *BIRC5* ⇔ *NEK2* ⇔ *TOP2A* ⇔ *CDKN3* ⇔ CCNB1 were found to be particularly strong and are confirmed by an underlying functional-interaction network as determined by querying the *STRING database*. Experimental evidence from putative homologues in other species suggests the existence of a protein-protein interaction network encompassing the majority of hub genes. Recently, Zhao et al. undertook an in-depth analysis of several datasets (GSE121248, GSE87630, GSE84598, and the TCGA-LIHC project) to identify potential biomarkers in HCC [75,76]. Several of the hub genes we detected (for example, *FEN1*, *TOP2A*, and *UBE2T*) were also identified as hub genes and potential biomarkers in these studies. Survival analysis showed that high expression of these genes, particularly *FEN1*, is associated with worse outcome [75]. Since we detected these hub genes in endothelial cells, we hypothesise that TEC play a role in mechanisms involving for instance *FEN1*, *TOP2A* or *UBE2T*. The M16 module presents itself as related to mitochondrial functions and may attest to the activated cell metabolism reported in tumour endothelial cells [77]. The findings of the GSVA, namely a downregulation of immunological functions in TEC (*complement activation, alternative pathway*), are mirrored in module M15. Thus, the M15 module may reflect EC’s impaired immunological function and their role in the cancer hallmark “Evading immune destruction”. The identification of 9 hub genes, for example, *CXCL12* and *F8*, highlights these genes’ role in functions associated with this module. Interestingly, no functional interactions have been found in the *STRING database*. The strong co-expression in these modules, for example *ANAPC1* ⇔ *TXND17*, *COA3* ⇔MEA1, or *PHKA1* ⇐ *MYO9A* may point towards novel, functionally relevant protein-protein interactions. Somewhat surprisingly, *CXCL12*, a chemokine with an important role in angiogenesis [78], was found to be a hub gene in a module negatively associated with TEC.

With an enrichment of the GO terms *Cell matrix adhesion* and *Basement membrane organisation*, the M18 module may display the “reorganisation of the basement membrane” and “adhesion” properties during angiogenic activation of endothelial cells [77]. No different eigengene expression was observed between NEC and TEC. Functions of angiogenic activated endothelial cells (for example, *Vascular endothelial growth receptor signalling pathway*, *endothelium development*, *regulation of angiogenesis*) characterise the module M14, highlighting this module’s role in angiogenesis and confirming the feasibility of our data. Interestingly, however, this module’s eigengenes were downregulated in *ENG*${}^{-}$ TEC, which may point towards a dedifferentiation of tumour endothelial cells. Finally, the enrichment of terms describing immunological functions in module M8 mirrors the findings of the GSVA concerning downregulation of immunological functions in TEC. TEC’s decrease of responsiveness to pro-inflammatory stimuli is well known and has been coined “tumor endothelial anergy” [62]. The association with positive regulation of immunological functions (for example *Positive regulation of cytokine production*, and *Positive regulation of leukocyte cell–cell adhesion*), combined with a decreased expression of the module eigengene in TEC highlights again their role in the *cancer hallmark* “Evading immune destruction”. The strong co-expression between the hub genes of the modules M14 and M8 may indicate novel, potentially relevant protein-protein interactions.

As a further result, querying the *Drug–Gene Interaction database* identified several potentially druggable genes, for example, *FEN1*, *BIRC5* and *NEK2*. *FEN1* is essential in maintaining genome stability, replication and repair [79,80]. It plays a major role in DNA replication and protection against apoptosis and the development of drug resistance [81], has been suggested as a potential target in a variety of cancers [82], and has also been identified as a potential hub gene in hepatocellular carcinoma [75,83]. *BIRC5* which is secreted into the microenvironment [84], acts as an apoptosis inhibitor [85] and may play a significant role in the apoptosis-resistant phenotype observed in TEC [20]. Therefore, our results may point towards a TEC mediated protection against apoptosis for the tumour cells. BIRC5 can be targeted by several drugs, for example, erlotinib, lapatinib, trastuzumab, or 5FU. The SEARCH trial investigated the efficacy of a combination of sorafenib and erlotinib but failed to reach the primary endpoint [86]. Lapatinib, a small-molecule inhibitor of *ERBB1*/*ERBB2* tyrosine kinases, has been shown to inhibit *BIRC5* expression via the *ERB2* signalling [87]. This inhibition, however, seems to take place at a posttranscriptional rather than transcriptional level [87]. Trastuzumab, which is primarily used in breast cancer [88], interacts via the PI3K pathway with BIRC5 [89] and shows some effectiveness [90]. Several drugs directly targeting *BIRC5* are under investigation, employing five different modes of action: disruption of BIRC5 interactions, inhibition of homodimerisation, decrease gene expression, degradation of *BIRC5* mRNA, and usage in immune therapy [64]. For example, shepherdin disrupts the binding of BIRC5 to HSP90, resulting in anti-cancer activity [91]. A 5-desflavacin analogue disrupts BIRC5-SMAC interactions [92]. Molecular docking studies identified other BIRC5-SMAC interrupting agents such as withanone or piperine derivates [93,94]. Recently it has been demonstrated that PZ-6-QN, a BIRC5-SMAC interaction disruptor, showed promising anti-cancer activity [95]. Taken together, these data indicate that the disruption of BIRC5 interactions may hold promise in cancer therapy. Interestingly, the M1 module contained only *SMAC*, but not *HSP90*. *SMAC* was not identified as hub-gene in the M1 module. *NEK2* plays a role in developing drug resistance against various anti-cancer drugs, including 5-fluorouracil and sorafenib [69,96,97]. A potential role of *NEK2* in resistance development against lenvatinib and other anti-angiogenic drugs has not been investigated yet. NEK2 has been proposed as a promising target in cancer therapy [98,99], and various drugs are currently under development. As with BIRC5, these drugs employ several modes of action, namely silencing mRNA expression using siRNA, blocking the ATP binding site, and interruption of protein-interaction [99]. Several small molecular compounds disrupt the interaction HEK-NEK1 resulting in NEK2 degradation and cell death. [100]. In our results, we found *HEC1* to be a member of the M1 module. However, as with *SMAC*, *HEC1* was not classified as hub-gene. Other functionally relevant genes, for example, *FEN1*, *NEK2*, *TOP2A*, *CCNB1* or *CDKN2*, displayed greater connectivity and co-expression strength within the module. Further, experimental evidence in other species indicates that some of these observed co-expression patterns may indicate underlying protein-protein interaction networks. According to the centrality-lethality theorem, genes with high connectivity are more likely to be therapeutic targets than genes with low connectivity [27]. We interpret these findings so that the reported co-expression networks could be utilised to identify potential two types of new therapeutic targets, namely among the hub genes themselves and protein-protein interactions associated with strong co-expression values.

Finally, we present an easy-to-implement framework, consisting of identifying differentially perturbed pathways via Gene Set Variation analysis, followed by a co-expression network-based determination of key modules and potential therapeutic targets. While this study successfully identifies differentially expressed genes, perturbed pathways, relevant gene modules and potential therapeutic targets and provides a blueprint for similar questions, it has several limitations. Although unique in study size and design, GSE51401 is a non-recent microarray-based dataset; therefore, it should be validated using newer RNASeq data. Second, the analysis has been done in silico only and requires, therefore, validation, as it should be done with all bioinformatical analyses. Preferably, this should be a combination of proteomic analysis and functional assays. Third, the analysis is restricted to one tumour entity and should be further validated in other tumour entities to allow more general conclusions. It would also be of interest to carry out pan-cancer endothelial profiling to characterise similarities and differences between endothelial cells from different tumour entities.

## 5. Conclusions

This study systematically investigated the differences between normal and tumour endothelial cells in hepatocellular carcinoma on gene expression level. Gene Set Variation Analysis showed a gene expression profile of tumour endothelial cells characterised by upregulated *evasion from growth suppressors* pathways, and downregulated *tumour-promoting inflammation* and *resisting cell death* pathways. Weighted Gene Co-expression Network Analysis identified several modules strongly associated with cell origin and cell activation. These modules were characterised by GO terms associated with cell proliferation, mitochondrial metabolism and immune functions. Cell proliferation and mitochondrial metabolism associated modules were upregulated, immune function associated modules were downregulated in TEC. Determination of hub genes revealed several druggable hub genes, such as FEN1, BIRC5, and NEK2, which may lead to new therapeutic approaches targeting endothelial cells. Finally, other researchers might use this framework as a blueprint when tackling similar questions in other tumours.

## Figures and Tables

**Table 1:** Characteristics of the study cohort. Numbers before the slash represent the number of used samples, numbers after the slash represent the number of total samples.

Gender	n	Median Age (Years)	Non-Tumour Endothelial Cells (NEC)		Tumour Endothelial Cells (TEC)	
			ENG^−^	ENG^+^	ENG^−^	ENG^+^
F	3	64	3/3	2/2	2/3 ^1^	2/2
M	13	52	12/13 ^2^	6/6	12/13 ^3^	4/6 ^4^
Total	16	53	15/16	8/8	14/16	6/8

^1^ Sample GSM1244757 (from Patient 8) was excluded due to unequal hybridization, as detected in spatial distribution plots. ^2^ Sample GSM1244761 (from Patient 9) was excluded due to excessive spans of the relative log expression (RLE) distribution and the normalized unscaled standard error (NUSE) distribution. ^3^ Sample GSM1244763 (from Patient 9) was excluded due to excessive distance to the other samples in the PCA as well as an excessive NUSE. ^4^ Sample GSM1244737 (from Patient 3) was excluded due to excessive distance to the other samples in the PCA. Sample GSM1244746 (from Patient 5) was excluded due to excessive spans of the relative log expression (RLE) distribution and the normalized unscaled standard error (NUSE) distribution.

**Table 2:** Potentially drugable hub genes in network modules positively associated with cell origin or angiogenic activation and the respective drugs.

Gene Symbol	Gene Name	Drug	PMIDs
**Module: M1**			
BIRC5	baculoviral IAP repeat containing 5	UCN-01, STAUROSPORINE, ROMIDEPSIN, VALDECOXIB, ERLOTINIB, OPRELVEKIN, VORINOSTAT, IMATINIB, TRASTUZUMAB, PACLITAXEL, FLUTAMIDE, CALCITONIN, GATAPARSEN, DEXAMETHASONE, ROFECOXIB, PLICAMYCIN, IRINOTECAN HYDROCHLORIDE, REGRAMOSTIM, EPIRUBICIN, TRETINOIN, SULINDAC, CARBOPLATIN, AMMONIUM TRICHLOROTELLURATE, DOCETAXEL, RESVERATROL, OMACETAXINE MEPESUCCINATE, METHOTREXATE, DOXORUBICIN, EPOETIN ALFA, LAPATINIB, INDOMETHACIN, CAMPTOTHECIN, PRASTERONE, GENISTEIN, FLUOROURACIL, RESERPINE, BERBERINE	15255949, 17255535, 14767553, 16707021, 17047074, 14734714, 16951239, 16254145, 23204226, 16452223, 15347474, 16211241, 15735703, 16222118, 16787583, 14627349, 17124180, 15956246, 16403261, 16608080, 14587026, 16950207, 16889755, 14729643, 15837718, 17270149, 15854289, 15670151, 16211302, 17112829, 16461558, 17375591, 15067352, 17968851
CCNB1	cyclin B1	KENPAULLONE, PROTOAPIGENONE, SELICICLIB	21080703
CDKN3	cyclin-dependent kinase inhibitor 3	PHA-793887, RONICICLIB, AZD-5438, AT-7519	
FEN1	flap structure-specific endonuclease 1	TYRPHOSTIN 23, APOMORPHINE HYDROCHLORIDE HEMIHYDRATE, STREPTONIGRIN, ISOTHYMONIN, CALMIDAZOLIUM CHLORIDE, PIRARUBICIN, MYRICETIN, PROTOPORPHYRIN DISODIUM, CIANIDANOL, DEOXIEPINEPHRINE, TRANSPLATIN, SLAZINIC ACID, OXIDOPAMINE HYDROCHLORIDE, PENTABROMOPHENOL, AMINODIMETHOXYQUINAZOLINYLPIPERAZINE, 4-CHLOROMERCURIBENZOIC ACID, ELLIPTECINE, PINAFIDE, HYDROXYZINE PAMOATE, CLINAFLOXACIN, SENNOSIDE B, 3-O-METHYLQUERCETIN, PHENYLSTIBONIC ACID, PURPUROGALLIN, IDARUBICIN, CLOSANTEL, OXOPURPUREINE, THUNBERGINOL B, DIOTYROSINE, TYRPHOSTIN 51, DAPHNETIN, TETRAIDOFLUORESCEIN, EBSELEN, MITONAFIDE, QUINACRINE, LEVODOPA, STICTIC ACID, DEMECLOCYCLINE, DOXYCYCLINE, ATHRAQUINONES A, AMARANTH, THIMEROSAL, CEPHALOCHROMIN, HAEMATOXYLIN, METHOXSALEN, COUMESTROL, AURINTRICARBOXYLIC ACID, GW305074X, FERROUS FUMARATE, FERROUS GLYCINE SULFATE, GOSSYPOL, NOREPINEPHRINE, CETYLPYRIDINIUM BROMIDE, BENSERAZIDE HYDROCHLORIDE, METHACYCLINE HYDROCHLORIDE, SURAMIN, QUERCETIN, ASTERRIC ACID, METHYLENE BLUE, EMODIN, LUTEOLIN, SANGUINARINE SULFATE, RHEIN, METHYLDOPA (RACEMIC), ELLAGIC ACID, HOMIDIUM, DEPHOSTATIN, HOMIDIUM BROMIDE, EPINEPHRINE, AMINACRINE HYDROCHLORIDE, DITHIAZANINE, ACRIFLAVINE, CARMINE, AMENTOFLAVONE, DEQUALINIUM, LAVENDUSTIN C, TOLONIUM CHLORIDE, PROTOPORPHYRIN, HEXAMETHYL PARAROSANILINE, TAXIFOLIN, ALEXIDINE HYDROCHLORIDE, PICEATANNOL, METHYLTHIONINIUM CHLORIDE, OXOGLAUCINE, FAGARONINE, ACID BLUE 129, MORIN, DAUNORUBICIN HYDROCHLORIDE, CEFSULODIN SODIUM, HISPIDIN, METHYLDOPA, PAMOIC ACID, FRAXETIN, LOMOFUNGIN, ISOKAEMPFERIDE, NORDIHYDROGUAIARETIC ACID, EUPAFOLIN, FURAMIDINE, SANGUINARIUM CHLORIDE, MITOXANTRONE, HYCANTHONE, PYRONIN Y, CHARTREUSIN, CARMINIC ACID, 2-METHOXY-1, 4-NAPHTHOQUINONE, ERBSTATIN, MITOXANTRONE HYDROCHLORIDE, BAICALEIN, CATECHOL, PURPURIN, OXYTETRACYCLINE, DOPAMINE, INDOCYANINE GREEN	3319774, 20622253
NEK2	NIMA related kinase 2	ADAVOSERTIB, HESPERADIN, R-406, DACTOLISIB, PAZOPANIB, DOVITINIB, FOSTAMATINIB, GW441756X, TAE-684, CENISERTIB, GW843682X, ILORASERTIB, PALBOCICLIB, CYC-116, SP-600125, GSK-579289A	19035792, 26516587
TOP2A	topoisomerase (DNA) II alpha	DOXORUBICIN, BECATECARIN, C-1311, CARINATIN G, ETOPOSIDE, AMSACRINE, PODOFILOX, VALRUBICIN, EPIRUBICIN, IDRONOXIL, AMONAFIDE, 13-DEOXYDOXORUBICIN, AMRUBICIN HYDROCHLORIDE, HYDROQUINONE, DIAZIRINE, LUPEOL, DOXORUBICIN HYDROCHLORIDE, UNGEREMINE, DAUNORUBICIN HYDROCHLORIDE, SPARFLOXACIN, ANNAMYCIN, ELSAMITRUCIN, LOMEFLOXACIN, NORFLOXACIN, DACTINOMYCIN, FLEROXACIN, LUCANTHONE, AMRUBICIN, VOSAROXIN, TENIPOSIDE, DAUNORUBICIN, MITOXANTRONE, DAUNORUBICIN CITRATE, DEXRAZOXANE, BETULIN, IDARUBICIN, KAEMPFERITRIN, GANCOTAMAB, BERUBICIN HYDROCHLORIDE, ELINAFIDE, ETOPOSIDE PHOSPHATE, MYRICETIN, DEMETHYLZEYLASTERONE, FRANGULIN B, IDARUBICIN HYDROCHLORIDE, PACLITAXEL, FLUOROURACIL, SECAUBRYOLIDE, MITOXANTRONE HYDROCHLORIDE, GENISTEIN, QUERCETIN, HURATOXIN, FISETIN, DIGITOXIN, MOXIFLOXACIN, PEFLOXACIN, TROVAFLOXACIN, ENOXACIN, DECLOPRAMIDE, CIPROFLOXACIN, OFLOXACIN, ELLIPTECINE, ADRIAMYCIN, 4’-O-ACETYLPATENTIFLORIN B, MAKALUVAMINE E, DIPHYLLIN, SIMOCYCLINONE D8, BANOXANTRONE, FINAFLOXACIN, MAKALUVAMINE C TFA SALT, CAMPTOTHECIN, MAKALUVAMINE A, MAKALUVAMINE F, ALDOXORUBICIN, LYCOBETAINE, VINCRISTINE, TRICITRINOL B, OLEANDEROLIDE	26211460, 17089011, 11752352, 17578914, 17010609, 20170164, 22276998, 21388138, 17351394, 17016621, 16377807, 16309315, 16271071, 8823806, 8870683, 23711769, 22867019, 25466187, 23968711, 23920485, 11678653, 25003995, 23566520, 21644529, 18258442, 25941559, 20863598, 22364746, 25922181, 23353750, 24931277, 24012683, 23360284, 20006518, 26216018, 17361331, 17514873, 9426516, 9485461, 9494516, 16759114, 25945730, 19691293, 24326278, 22620261, 26264845, 19783445, 26291037, 19386396, 24334150, 21435753, 25815139, 22867097, 25799376, 25800514, 25240702, 24775914, 18816045, 26292628, 24507920, 24095018, 1311390, 11473732, 17911018, 19155103, 25626146, 22537681, 19725581, 11006484, 11716434, 10691026, 1322791, 8519659, 8632768, 1845848, 10783066, 16061385, 1331331, 1334447, 16019763, 16234514, 17639997, 14728934, 17658777, 24054489, 15833037, 11754608, 22014547, 7756657, 3015015, 1323952, 9169823, 10487533, 17628745, 16480143, 8702194, 22260166, 1963303, 6380596, 11004693, 9631585, 10451375, 11278845, 18687447, 11046078, 12911317, 10194547, 17115008, 11179439, 17652819, 11984069, 11332155, 10203104, 12034365, 11836027, 8036155, 10523799, 22014228, 21880496, 7769390, 8759170, 20561793, 17139284, 17016423, 20596674, 20802486, 18471102, 10089819, 2847647, 22119124, 8691207, 17340571, 25808831, 11205246, 25312684, 23266176, 21761866
TTK	TTK protein kinase	HESPERADIN, BAY-1217389, FOSTAMATINIB, BAY-1161909	19035792, 26516587
UBE2T	ubiquitin conjugating enzyme E2T	MK-2206	
**Module: M14**			
HSPG2	heparan sulfate proteoglycan 2	HALOPERIDOL, PALIFERMIN, CYCLOSPORINE	27023437, 10593896, 16989989, 14753849, 9788974, 14974815
PEAR1	platelet endothelial aggregation receptor 1	ASPIRIN, CLOPIDOGREL, PRASUGREL, TICAGRELOR	23392654, 23859572, 28820077, 26962983, 27937053
ERG	v-ets avian erythroblastosis virus E26 oncogene homolog	DOFETILIDE, SOTALOL HYDROCHLORIDE, AZD1305, NERISPIRDINE, IDARUBICIN HYDROCHLORIDE, MITONAFIDE, N-ACETYLASPARTIC ACID, GUANIDINE HYDROCHLORIDE, DALFAMPRIDINE, AZD7009, AMIODARONE HYDROCHLORIDE, MEDROXYPROGESTERONE ACETATE, HOMIDIUM BROMIDE, DAUNORUBICIN HYDROCHLORIDE, PERGOLIDE MESYLATE, 1, 4-DIMETHOXYANTHRAQUINONE, TEDISAMIL	

## Data Availability

The data analyzed in this study are openly available in Gene Expression Omnibus, accession number GSE51401.

## Supplementary Materials

The following are available online at https://www.mdpi.com/article/10.3390/cancers13081768/s1, Figure S1: The “Cell Cycle” Pathway, Figure S2: The “Retinoblastoma gene in cancer” Pathway, Figure S3: The “MAPK signalling Pathway”, Figure S4: The “Chemokine signalling pathways” Pathway, and Figure S5: The “Apoptosis” pathway. Table S1: LIMMA results and connectivity of the genes, Table S2: Results of the GSVA between contrasts, Table S3: Results of the Term Enrichment Analysis of the single modules, and Table S4: Results of the drug gene interaction database query.

## Abbreviations

The following abbreviations are used in this manuscript:

DGIdb	*Drug–Gene Interaction database*
NEC	endothelial cells derived from non-tumour tissue (liver sinusoidal, macrovascular, and other endothelial cells)
TEC	endothelial cells derived from tumour tissue (liver sinusoidal, macrovascular endothelial cells, and other endothelial cells)
LSEC	Liver sinusoidal endothelial cells
LEC	Liver endothelial cells (liver sinusoidal, macrovascular, and other endothelial cells)
HCC	Hepatocellular carcinoma
log2FC	logarithm of the fold change to the base 2
WGCNA	Weighted Gene Network Analysis
GSEA	Gene Set Enrichment Analysis
GOTEA	GO Term Enrichment Analysis
GSVA	Gene Set Variation Analysis
GOTSSA	GO Term Semantic Similarity Analysis
FI	Functional interactions
DEG	differentially expressed gene
5FU	5-fluorouracil
