# Supplementary material for: Systematic Analysis of the Transcriptome Profiles and Co-Expression Networks of Tumour Endothelial Cells Identifies Several Tumour-Associated Modules and Potential Therapeutic Targets in Hepatocellular Carcinoma
Source: Cancers (Basel). 2021 Apr 7;13(8):1768. doi: 10.3390/cancers13081768 (PMC8067977; doi:10.3390/cancers13081768)
Supplement: Supplementary file 1 [file cancers-13-01768-s001.zip › supply/cancers-1133034-supply.pdf]

Systematic analysis of the transcriptome profiles and co-expression networks of tumour endothelial cells identifies several tumour-associated modules and potential therapeutic targets in hepatocellular carcinoma - Supplementary Material

Mohr, T.; Katz, S.; Paulitschke, V.; Aizarani, N.; and Tolios, A.

Supplementary Tables

Table S1: **LIMMA results and connectivity of the genes.** Columns indicate: PROBEID: Affymetrix probe ID, ACCNUM: GenBank accession number(s), ENTREZID: Entrez gene ID(s), ENSEMBL: Ensemble gene ID(s), ENSEMBLTRANS: Ensembl transcript ID(s), REFSEQ: RefSeq gene ID(s), SYMBOL: canonical gene symbol, GENENAME: Gene name, UNIPROT: Uniprot protein ID, logFC: log2 fold change, AveExpr: average expression, t: t-statisics, P.Value: p-value, adj.P.Val: adjusted p-value (Benjamini Hochberg), B: log odds, module: assigned module, kTotal: the total connectivity, and kWithin: konnectivity within the module.

Table S2: **Results of the GSVA between contrasts.** Columns indicate: vresion: pathway source, wpid: pathway identification, org: species, name: name of the pathway, hallmark: assoicated hallmark of cancer, logFC: change between GSVA scores, AveExpression: mean between two GSVA scores, t: t-statisics, P.Value: p-value, adj.P.Val: adjusted p-value (Benjamini Hochberg), B: log odds.

Table S3: **Results of the Term Enrichment Analysis of the single modules.** Columns indicate: ID: Term ID, Description: Term name, GeneRatio: Ratio of genes present to genes in the pathway, BgRatio: Ratio of genes annotated with the term to the total number of genes, pvalue: p-value, p.adjust: adjusted p-value, qvalue: q-value, geneID: HGNC symbols of genes differentially expressed, Count: number of genes differentially expressed, cluster: cluster according to the similarity analysis and umap, and parent and parentTerm: parent term according to the similarity analysis.

Table S4: **Results of the drug gene interaction database query.** Columns indicate: SYMBOL: Gene symbol, GENENAME: gene name, Drug: drug, Source: source for the drug-gene interaction, PMIDs: pub med ids of the publications, $kWithin_{norm}$: Normalized connectivity within the module.

Supplemntary Figures

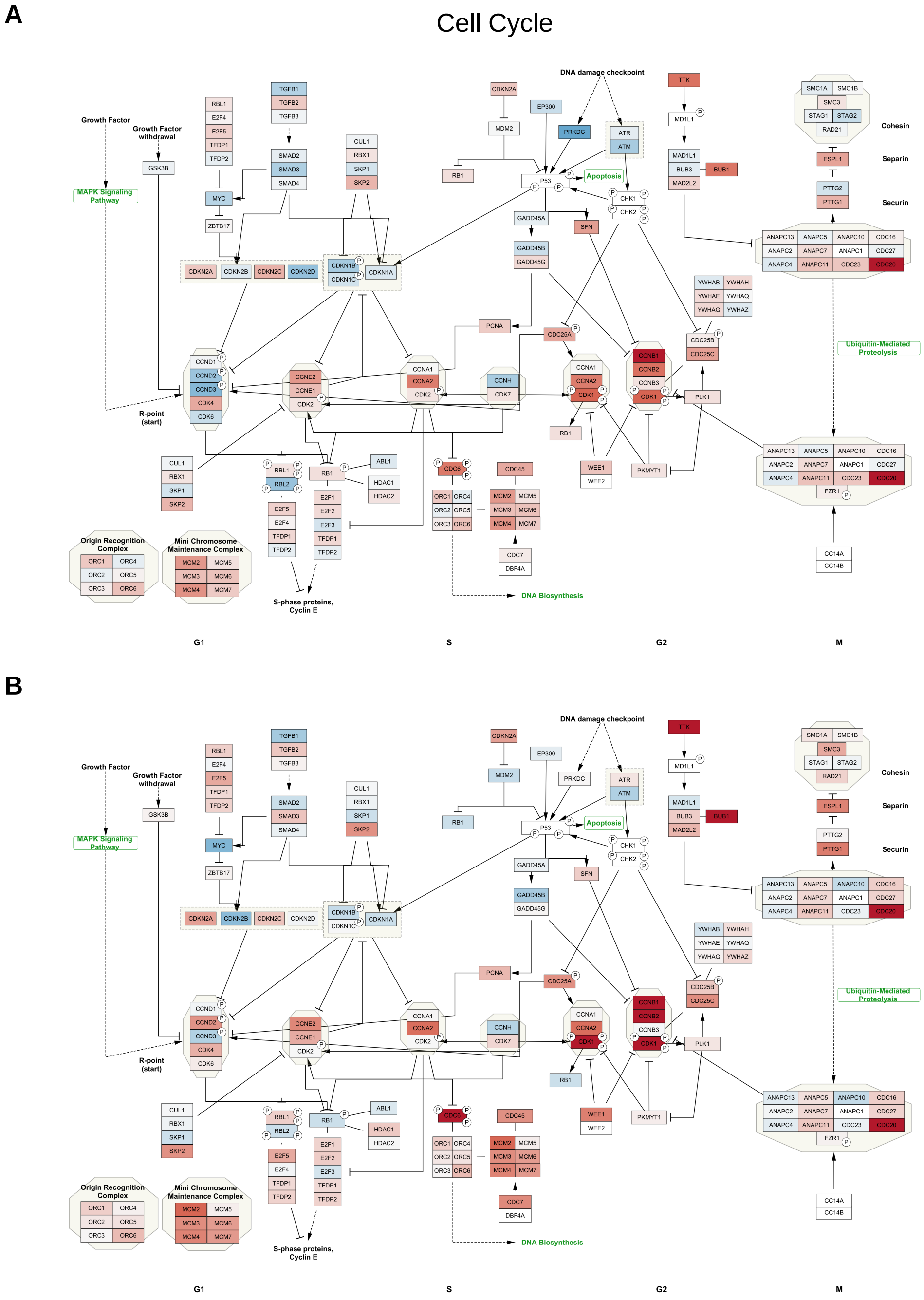

Figure S1: **The "Cell Cycle" Pathway.** (A) ENG$^-$ TEC compared to ENG$^-$ NEC. (B) ENG$^+$ TEC compared to ENG$^+$ NEC. Coloration indicates regulation, red means upregulation, blue downregulation in TEC.

A

Retinoblastoma Gene in Cancer

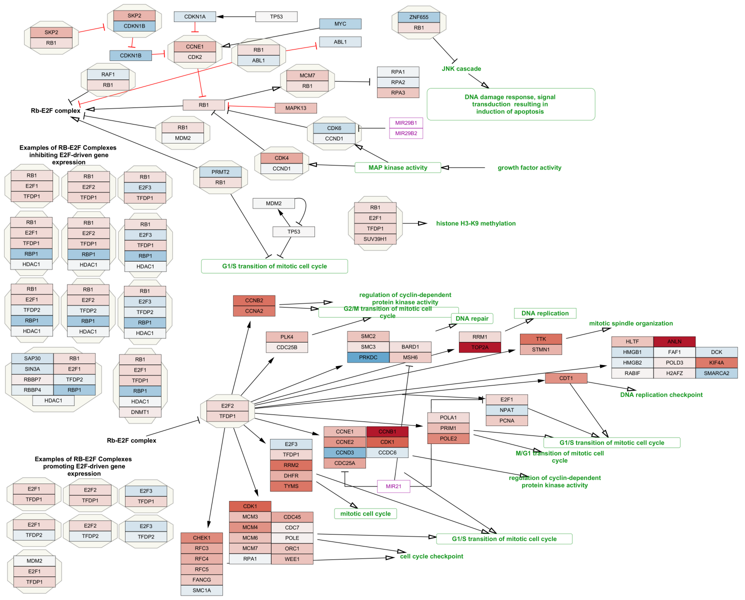

B

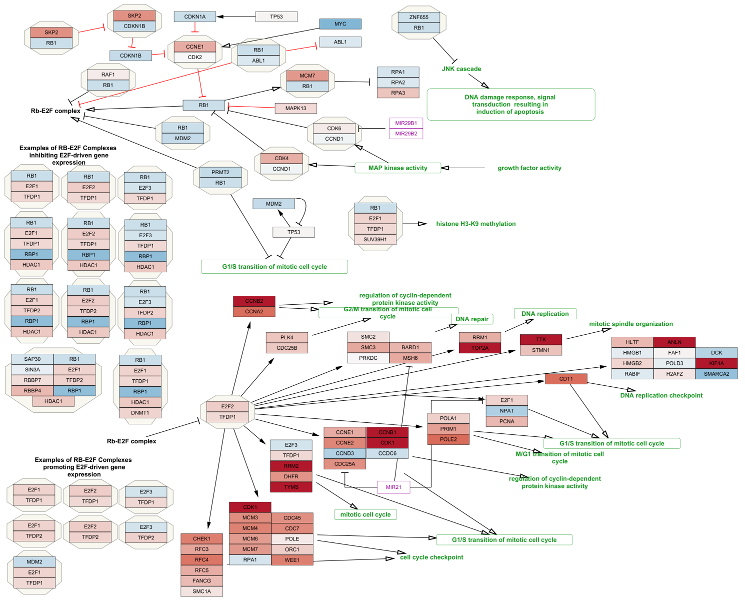

Figure S2: **The "Retinoblastoma gene in cancer" Pathway.** (A) ENG$^-$ TEC compared to ENG$^-$ NEC. (B) ENG$^+$ TEC compared to ENG$^+$ NEC. Coloration indicates regulation, red means upregulation, blue downregulation in TEC.

A

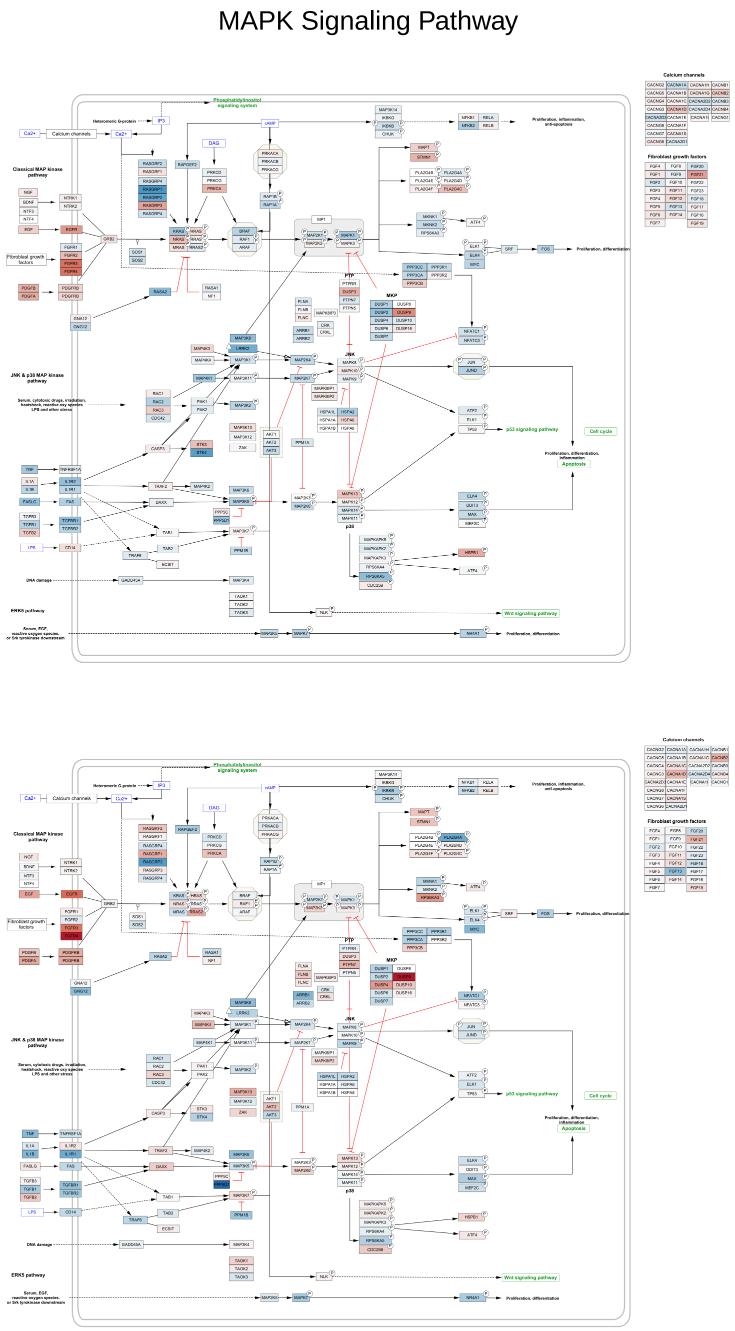

B

Figure S3: **The "MAPK signalling Pathway".** (A) ENG⁻ TEC compared to ENG⁻ NEC. (B) ENG^{+} TEC compared to ENG^{+} NEC. Coloration indicates regulation, red means upregulation, blue downregulation in TEC.

A

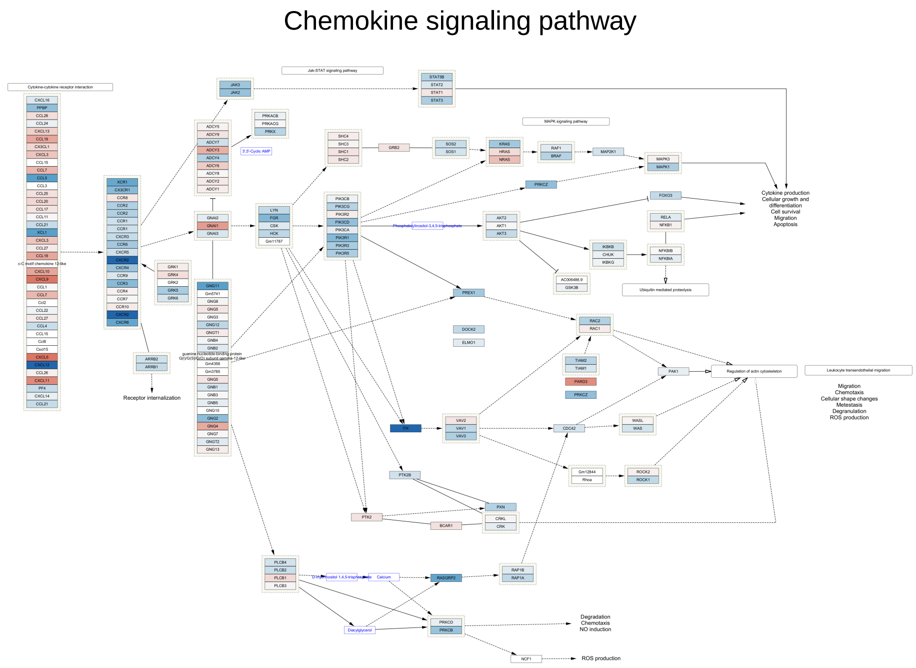

B

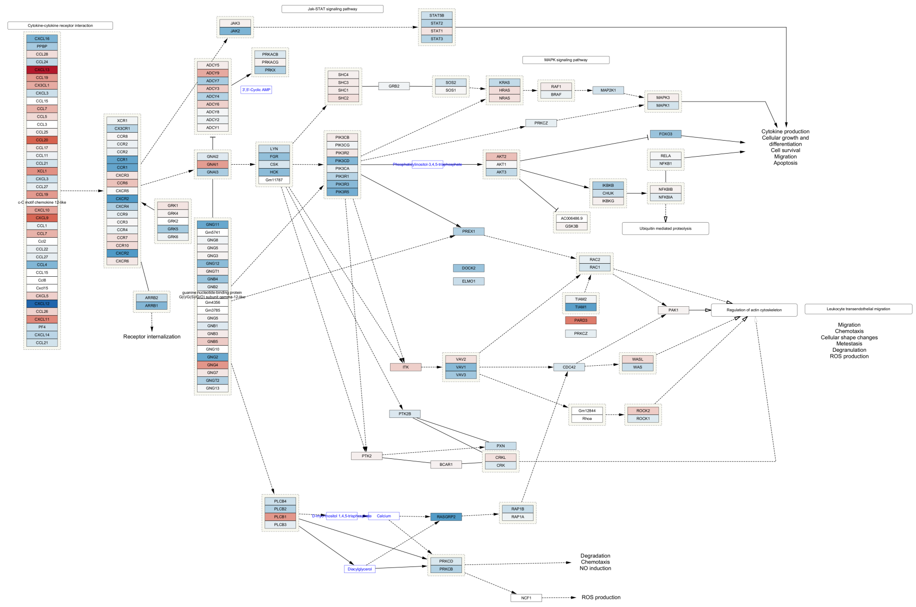

Figure S4: **The "Chemokine signalling pathways" Pathway.** (A) ENG⁻ TEC compared to ENG⁻ NEC. (B) ENG$^+$ TEC compared to ENG$^+$ NEC. Coloration indicates regulation, red means upregulation, blue downregulation in TEC.

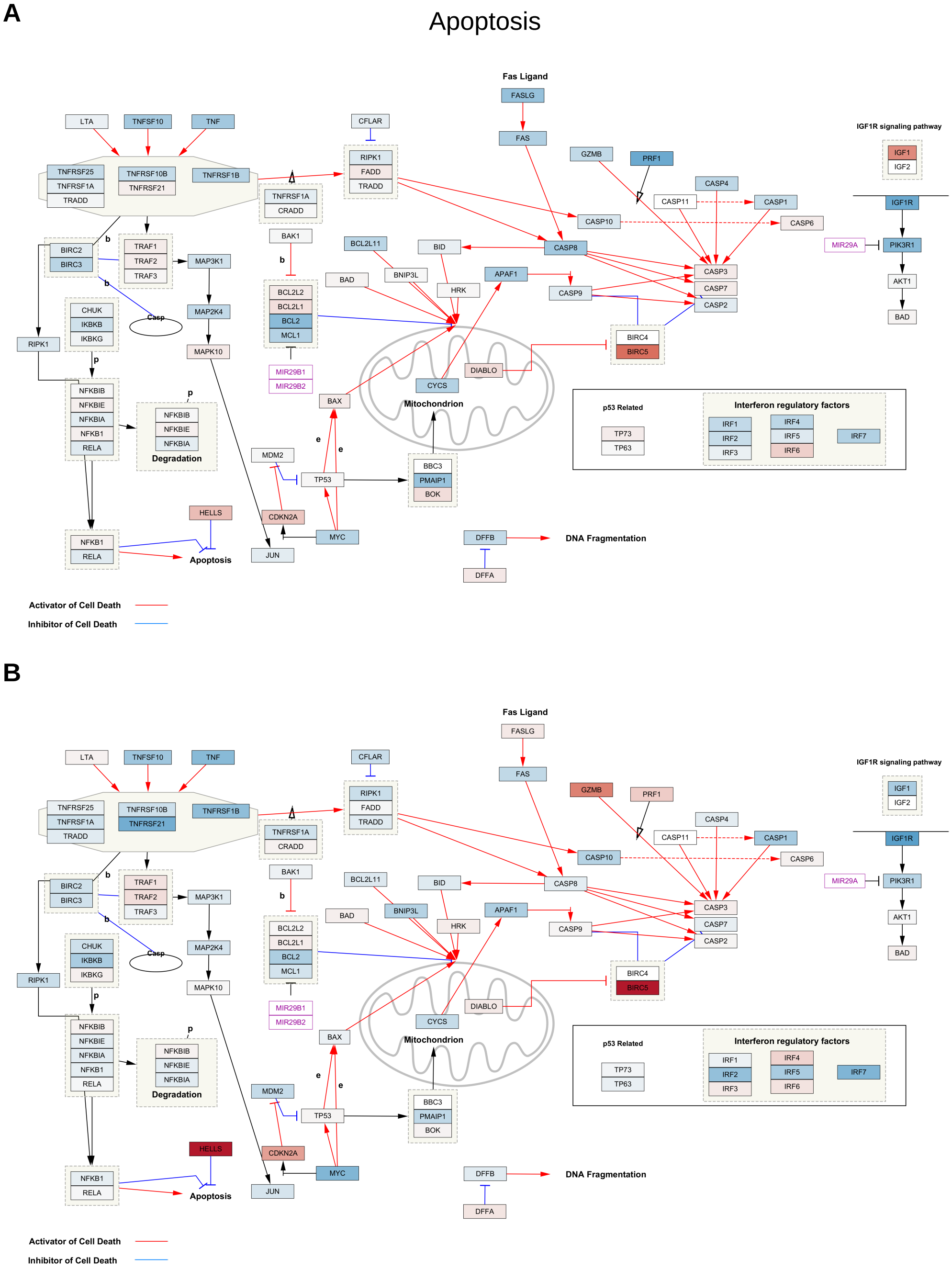

Figure S5: **The "Apoptosis" pathway.** (A) ENG⁻ TEC compared to ENG⁻ NEC. (B) ENG$^+$ TEC compared to ENG$^+$ NEC. Coloration indicates regulation, red means upregulation, blue downregulation in TEC.
